# Use of GP services by patients with schizophrenia: a national cross-sectional register-based study

**DOI:** 10.1186/s12913-015-0719-1

**Published:** 2015-02-18

**Authors:** Øystein Hetlevik, Magne Solheim, Sturla Gjesdal

**Affiliations:** Department for Global Public Health and Primary Care, University of Bergen, N-5020 Bergen, Norway

**Keywords:** Schizophrenia, Mental health, Primary care, General practice, Healthcare services

## Abstract

**Background:**

Reform of health services has given primary care facilities increased responsibility for patients with serious mental disorders (SMD). There has also been a growing awareness of the high somatic morbidity among SMD patients, an obvious challenge for general practitioners (GPs). The aim of this study was to assess the utilisation of GP services by patients with schizophrenia.

**Methods:**

The Norwegian list patient system is based on fee-for-service (FFS). For each contact, the GPs send a claim to National Health Insurance detailing the diagnosis, the type of contact, procedures performed, and the personal identifier of the patient. In this study complete GP claims data from 2009 for schizophrenia patients aged 25–60 years were used to assess their utilisation of GP services. Regression models were used to measure the association between patient, GP and practice characteristics, with FFS per patient used as a measure of service utilisation. Data on patients with diabetes (DM) and population means were used for comparison.

**Results:**

The mean annual consultation rate was 5.0 and mean FFS was 2,807 Norwegian Kroner (NOK) for patients diagnosed with schizophrenia. Only 17% had no GP consultation, 26.2% had one or two, 25.3% had three to five, and 16.1% more than five consultations. GPs participated in multidisciplinary meetings for 25.7% of these patients. In schizophrenia patients, co-morbid DM increased the FFS by NOK 1400, obstructive lung disease by NOK 1699, and cardiovascular disease by NOK 863. The FFS for schizophrenia patients who belonged to a GP practice with a high proportion of mental health-related consultations increased by NOK 115 per percent point increase in proportion of consultations. Patients with schizophrenia living in municipalities with < 10,000 inhabitants had a mean increase in FFS of NOK 1048 compared with patients living in municipalities with > 50,000 inhabitants. Diagnostic tests were equally or more frequent used among patients with schizophrenia and comorbid somatic conditions than among similar patients without a SMD.

**Conclusion:**

This study showed that most patients diagnosed with schizophrenia had regular contact with their GP, providing opportunities for the GP to care for both mental and somatic health problems.

## Background

Over the last few decades, reforms have resulted in primary care services having increased responsibility for patients with serious mental disorders (SMD) though there are important variations between countries [1,2]. In Norway, the municipal health services have been strengthened by recruitment of a large number of mental health care workers, while the resources for general practitioner (GP) services have increased only slightly [3]. In 2001 a national list patient system was introduced with the main aim of improving the continuity of care for patients in the community with chronic disorders.

GPs are the first line of contact for patients with mental health problems. GPs often make a tentative diagnosis and normally refer patients to specialised care when a SMD is considered [4]. Findings differ concerning the role of GPs in the further treatment of patients with SMD, but GPs have contact with a large proportion of these patients over time [5-7]. For instance, in Norway, GPs prescribe nearly 70% of all antipsychotic medications [8].

The high somatic morbidity among patients with SMD is now being addressed [9,10]. Patients with schizophrenia have a life expectancy nearly 25 years shorter than the population mean [11]. Smoking, obesity, and lack of physical activity are common among patients with SMD [12,13]. Antipsychotic drugs have negative metabolic effects [14], contributing to an increased prevalence of diabetes (DM) [15]. Cardiovascular and pulmonary diseases are more prevalent, but are often underdiagnosed [16]. Prevention, diagnosis and treatment of somatic diseases are sub-optimal among patients with schizophrenia [9,17-20].

While views and guidelines on the role of GPs in follow-up of SMDs differ [4,21,22], they are expected to provide good somatic health services to all their patients [4]. In a British study, patients with schizophrenia received fewer general medical checkups compared with other patient groups [18]. However, more recently a Dutch study indicated that the treatment of diabetes (DM) and chronic obstructive pulmonary diseases (COPD) among patients with SMD in general practice was comparable to the care given to other patients with the same diseases [23].

There is a need for a comprehensive approach to treatment of patients with SMD [24,25]. A good relationship with a GP and stable cooperation with the local healthcare services can improve social participation and quality of life, and increase survival among patients [2,22,24,26]. In surveys, health professionals agree on the positive effects of improved cooperation [25,27], but it is often difficult to achieve well-functioning cooperation in practice [6]. There are claims that Norwegian GPs are too little engaged with mental health problems in general and especially in the multidisciplinary cooperation required for patients with SMD [28], but there are limited data available.

The first aim of the study was to increase the knowledge about the contribution of GPs to the care of patients with SMD, by assessing how schizophrenia patients used GP services with respect to both mental and somatic health problems. The second aim was to study variations among GPs and their care for these patients using patient, GP, and GP practice characteristics as explanatory variables. For comparison, we used similar data on the total population and non-schizophrenia patients diagnosed with DM.

## Methods

A register-based, cross-sectional nationwide study of utilisation of general practice services in Norway in 2009 was conducted based on two national registers. *The Regular GP database* has information about all regular GPs contracted to municipalities and to the National Health Insurance (HELFO), including age and gender of the GPs, their patient list size, and the municipality in which they practice. This database also includes the identity of the patients on each list. A large portion of the reimbursement of Norwegian GPs is fee-for-service (FFS), paid by HELFO based on a GP tariff. *The KUHR database* contains all GP claims for FFS. For each patient-related contact, the GP sends a claim to HELFO along with the patient’s personal identity number. The claim includes codes from the GP tariff indicating the type of contact (consultation, home visit and brief contact with the office, by telephone or mail). There are also codes for laboratory tests. On-site measurements of glycosylated haemoglobin (HbA1c), ECG, and spirometry are used to diagnose or monitor somatic conditions and these codes are used as indicators of the individual GP’s practice. In addition, there are codes for the GP’s participation in a multidisciplinary meeting or communication by letter or telephone with municipal health or social services in relation to a defined patient. In this study we also used the specified code for consultation time > 20 minutes and a code for “talking therapy” in consultations for patients previously treated in specialised mental health care.

All claims also include a diagnosis according to the International Classification of Primary Care (ICPC) coding system. ICPC is organised in chapters corresponding to organ system and includes a chapter for psychological problems (P chapter), composed of defined symptoms and syndromes, of which schizophrenia is assigned the code P72.

### Study population

All Norwegian residents from 25 to 60 years of age and their GPs were included in the first part of the study (Table 1). The regression analyses using GP characteristics as explanatory variables were limited to patients with the same regular GP throughout 2009. Only GPs regularly practicing for at least 10 months in 2009 and their patients were included (N = 3220 GPs). The characteristics of the GPs included in the analyses are compared with all regular GPs in Norway in Table 2.

**Table 1:** **Use of GP services in Norway by patients diagnosed with schizophrenia compared with patients with diabetes and whole population**

**Patient groups**	**Schizophrenia**	**Diabetes**	**Total study population**
N	10,112	54,580	2,333,944
Proportion men (%)	61.9	59.0	51.1
Mean age (SD)	43.0 (9.7)	49.3 (8.6)	42.3 (10.1)
Number of years with same regular GP, median (interquartile range)^1^	5 (2–8)	8 (3–8)	8 (3–8)
***Frequencies and content of patient contacts***			
Proportion of patients with ≥1 consultation (%)	83.0	94.1	67.1
Mean number of consultations (SD)	5.0 (6.3)	5.7 (4.8)	2.6 (3.6)
Proportion of consultations lasting ≥20 min (%)^2^	19.0	37.7	31.1
Proportion of consultations with «talking therapy» (%)^2^	41.3	3.7	4.3
Proportion of consultations ≥20 min or with «talking therapy» (%)^2^	60.3	41.4	35.4
Proportion of consultations with any laboratory test taken (%)	74.0	74.4	50.6
Mean number of “short patient contacts”^3^ per patient (SD)	5.0 (8.7)	4.2 (5.2)	2.1 (3.9)
Mean number of home visits per patient (SD)	0.14 (1.59)	0.02 (0.96)	0.006 (0.26)
Proportion of patients that had taken:			
- HbA1c (%)	12.0	64.5	4.2
- ECG (%)	6.5	6.4	4.1
- Spirometry (%)	4.0	5.1	2.9
***Interdisciplinary activity by GPs***			
Proportion of patients with ≥1 multidisciplinary meeting, %	25.7	3.1	1.6
Mean number of multidisciplinary meetings per patient (SD)	0.65 (1.63)	0.06 (0.42)	0.03 (0.34)
Mean number of short communications^3^ between the GP and health- or social services per patient (SD)	2.9 (5.4)	0.37 (2.08)	0.13 (1.1)
Total service utilisation			
Annual fee-for-services per patient (NOK)^4^, mean(SD)	2807 (3956)	1943 (2439)	648 (1423)
***Comorbidity – proportion of patients also diagnosed with:***			
- Diabetes mellitus (%)	8.5	-	2.4
- Obstructive lung disease (%)	6.7	8.4	4.7
- Hypertension (%)	7.5	32.8	8.4
- Cardiovascular disease (%)	2.0	9.0	1.7

Data from 2009 on 10,112 patients in the age group 25–60 years diagnosed with schizophrenia compared with non-schizophrenia patients with diabetes (N = 54,580) and the total population (N = 2,333,944) registered with a regular GP.

^(1)^Maximum duration 8 years, since the list patient system was established in 2001. ^(2)^These fees are not allowed in combination, but both are representing long consultations, and therefore also shown combined. ^(3)^Telephone or letters. ^(4)^1€ = NOK 8.73 in 2009. GP, general practitioner; HbA1c. glycosylated haemoglobin; SD, standard deviation.

**Table 2:** **Characteristics of the GPs included in the regression analyses** ^*^ **compared with all regular GPs in Norway**

	**Included GPs**	**All regular GPs**
N	3220	4183
GP age, mean (IQR)	51 (42–57)	49 (39–57)
Proportion of male GPs, %	68,7%	65.3%
Proportion of GP approved specialist in general practice, %	69.0%	59.4%
List size, mean (IQR)	1219 (999–1482)	1191(938–1435)
Proportion of lists open for new patients, %	26.2%	30.9%
Number of patients with schizophrenia in the list population, mean (IQR)	3 (1–5)	3 (1–5)
Proportion of consultations (%) with a psychological diagnosis, mean (IQR)	9.8 (7.6–12.5)	9.8 (7.5–12.5)
Proportion of GPs (%) working in municipalities with:		
<10,000 inhabitants	25.7%	28.3%
10–20,000 inhabitants	16.2%	16.1%
20–50,000 inhabitants	23.1%	21.9%
>50,000 inhabitants	35.0%	33.7%

Data from 2009.

*Inclusion criteria for GPs, based on claims data from 2009: Fee for service claims for ≥ 10 months, ≥ 500 consultations, and having a list size above the lower limit of 500. IQR, interquartile range.

### Classification of patient groups

Based on the diagnoses used on GP claims from an extended time period of three years (2007–2009), 10,112 patients were classified with schizophrenia (ICPC code P72 used at least once), and 864 of these patients were also diagnosed with DM. For comparison, a DM group from the total population was constructed, and comprised 55,444 patients with at least two contacts labelled with codes T89 or T90 and who had no GP diagnosis of schizophrenia, assuming that the more common diagnosis of DM might be used with less precision among GPs.

### Outcome measurement

The annual total FFS per patient in 2009 was used as the main outcome measure. The FFS payments indicate the total use of GP services per patient, reflecting the number of consultations, short patient communications, home visits, medical procedures, and GPs’ participation in interdisciplinary meetings related to the patient.

### Explanatory variables

The patient variables used were age, gender, and time registered with the current GP. Variables indicating comorbidity were constructed using diagnoses in GP claims from 2007–2009 to identify patients with a diagnosis of obstructive lung disease (ICPC codes R95-96), hypertension (ICPC codes K85-87), or cardiovascular diseases (ICPC codes K74-76, K89-91). The GP variables used were age, gender, list size, free capacity in the practice list, and the size of the municipality in which the practice was based. In addition, for each GP, the proportion of all GP consultations with an ICPC diagnosis from the P chapter (psychological) were determined, as an indicator of each GP’s “mental health practice profile”. GPs qualified as specialists in family medicine have a higher fee per consultation compared with non-specialists; therefore, we adjusted for specialist status in the regression analysis.

### Statistical analysis

The *t*-test and Pearson chi-square test were used to compare patient groups. A p-value < 0.05 was considered significant. The distribution of FFS was markedly skewed to the right and a significant proportion of individuals had zero FFS. To account for these features a two-part model was used in the regression analysis (Table 3) [29]. This model is based on the fact that the expected FFS (per patient) is equal to the probability that FFS is positive multiplied by the conditional expected value of FFS, given that FFS > 0. For the first part we used logistic regression analysis to estimate the probability of FFS > 0, and for the second part (expected FFS given that FFS > 0) a gamma regression with log link was used. The same explanatory variables were included in both parts. The analyses were carried out using Stata 12 (Stata Corp., College Station, TX, USA) with the user written command tpm [30]. Another feature of the data was that individuals were clustered within GPs, so that the observations may not be independent. Therefore, cluster robust standard error estimation was used [31]. Results from the regression analyses are reported as average marginal effects [32]. The average marginal effect of a variable measures how much the expected FFS changes as the variables increase by one unit.

**Table 3:** **Association between the use of GP services and characteristics of patients and GP practices**

**Patient groups**	**Model 1**	**Model 2**	**Model 3**
	**Schizophrenia**	**Diabetes**	**Whole population**
	**(N =7,933)**	**(N = 44,732)**	**(N = 1,815,555)**
***Patient factors***			
Patient male	−492.62***	−437.84***	−365.04***
Patient age, years	−22.30***	2.40	0.67***
Year with the same regular GP	−46.42*	−35.45***	−19.50***
***Comorbidity***			
Diabetes mellitus	1398.68***	--	1118.31***
Obstructive lung disease	1699.17***	893.36***	665.70***
Hypertension	482.67**	253.51***	400.25***
Cardiovascular disease	863.14*	734.90***	862.16***
***GP and list characteristics***			
GP male	145.33	17.33	36.47***
GP age, years	- 5.21	−2.30	0.18
List size (per 100 patients)	−36.22*	−7.91	−4.52***
List open to new patients	−168.48	121.26**	11.27
GP’s proportion of consultations with a psychological diagnosis, per percentage point	114.68***	35.31***	19.04***
***Size of practice municipality***			
> 50,000 inhabitants	Ref	Ref	Ref
20–50,000 inhabitants	7.37	51.90	33.33***
10–20 000 Inhabitants	807.58***	205.11***	72.72***
< 10,000 inhabitants	1047.55***	97.38*	69.72***

Association between GP services utilisation estimated by fee-for-service (FFS) per patient in 2009 and characteristics of patients, GPs, GP practices, and practice municipality for patients aged 25–60 with schizophrenia or diabetes, and the whole population. Two-part regression models, presenting marginal effects in Norwegian Kroner (NOK), adjusted for being a specialist in family medicine (higher fees). For the population included in the regression analyses the mean annual FFS was 2,732 NOK for patients with schizophrenia, 1,963 NOK for patients with DM and 653 NOK for the whole population.

Data from 3220 Norwegian GPs, criteria for inclusion are given in Table 2.

*p < 0.05 **p < 0.005 ***p < 0.001.

### Approval

The Norwegian Data Inspectorate and the Norwegian Directorate of Health, as responsible for administration of the registers, approved the study. All patient data were anonymised.

## Results

### Use of GP services

In the age group 25–60 years, 10,112 patients (0.4%) had a diagnosis of schizophrenia (P72) in the GP claims. In Table 1, the use of GP services in 2009 among patients diagnosed with schizophrenia is compared with that of patients with DM (no schizophrenia) and of the whole population. The annual GP consultation rate for the schizophrenia group was 5.0. In 63.2% of these consultations, the GP used a main diagnosis from the psychological chapter in ICPC, compared with 14.3% in the total population. Among the patients with a diagnosis of schizophrenia, 17% had no GP consultations in 2009, 26.2% had one or two, 25.3% had three to five, and 16.1% more than five consultations. The GPs participated in at least one multidisciplinary meeting for 25.7% of the schizophrenia patients. For 53.8% of these patients the GPs reported at least one contact with municipal health or social services by mail or telephone. In 41.3% of the consultations in the schizophrenia group, “talking therapy” was used compared with 4.3% the total population.

The mean annual FFS among the patients with schizophrenia was 2,807 Norwegian Kroner (NOK) compared with 1,947 NOK in diabetic patients and 648 NOK in the total population.

### Comorbidity

According to the diagnoses used by the GPs, 8.5% (N = 864) of the patients with a diagnosis of schizophrenia also had a diagnosis of DM, compared with 2.4% in the total population aged 25–60 years (p < 0.001) (Table 1). In the schizophrenia group, 6.7% also had a diagnosis indicating obstructive lung disease, 7.5% cardiovascular disease, and 2.0% hypertension.

For patients with comorbid DM and schizophrenia, the mean consultation rate was 7.2 (standard deviation (SD), 6.9) compared with 5.7 (SD, 4.8) among DM patients without schizophrenia (p < 0.001); 70.5% had a HbA1c test during the study period, with an annual mean of 2.3 (SD, 2.5) tests per patient. For non-schizophrenia DM patients, 64.5% had a HbA1c test (p < 0.001), and the mean number of tests was 1.9 (SD, 2.0) (p < 0.001).

Among patients with both obstructive lung disease and schizophrenia, 22.4% had a spirometry test, compared with 22.8% among non-schizophrenia patients with obstructive lung disease (p = 0.77).

Among patients with a diagnosis of cardiovascular disease, an ECG was performed in 16.7% of those with schizophrenia and 18.7% in the others (p = 0.47). For patients with hypertension, an ECG was performed in 14.2% and 13.2%, respectively (p = 0.38).

### Variations in GP utilisation

Table 3 shows multivariate regression models estimating the associations between use of GP services indicated by FFS, patient, and GP characteristics, with separate analyses for patients with schizophrenia, DM, and the total population. Female patients diagnosed with schizophrenia had a 20% higher average marginal effect for FFS compared with male schizophrenia patients, all other variables being constant. Younger patients had higher FFS. The average marginal effect for FFS was not associated with GP age or gender.

In the mental health practice profile, a high proportion of psychological diagnoses in all consultations in a GP practice was associated with a higher use of GP services by patients diagnosed with schizophrenia. With a five percentage point increase, representing the inter-quartile range (Table 2), the marginal effect was 575 NOK, indicating a 20% increase in service utilisation. However, it was noted that belonging to this group of GPs was also associated with a higher utilisation of GP services generally.

A longer patient-GP relationship was associated with reduced FFS in all three models. Among patients with schizophrenia, comorbid DM increased the mean annual FFS by 1400 NOK. Comorbid obstructive lung disease or cardiovascular disease increased the mean FFS by 1699 NOK and 863 NOK, respectively. In the DM group, the respective figures were 893 and 734 NOK.

Patients with schizophrenia living in smaller municipalities had a higher FFS shown by a average marginal effect of NOK 1047 when comparing municipalities with < 10,000 inhabitants to municipalities with > 50,000 inhabitants.

## Discussion

### Main findings

In Norwegian general practice, the mean annual consultation rate for patients diagnosed with schizophrenia was 5.0 in 2009; 17% had no consultations in 2009, 26.2% had one or two, 25.3% had three to five, and 16.1% more than five consultations. The GPs participated in at least one multidisciplinary meeting for 25.7% of the patients with a diagnosis of schizophrenia. The total GP service utilisation indicated by the mean annual FFS expenditure per patient for patients diagnosed with of schizophrenia was 2,807 NOK compared with 1,943 NOK among the non-schizophrenia DM group, even although there was a 6-year older mean age in the DM group.

The use of diagnostic tests such as HbA1c, spirometry and ECG was equal or more frequent among patients with schizophrenia and comorbid somatic conditions, than among similar patients without a SMD.

### Strengths of the study

The main strength of a register-based study is the availability of complete and accurate data concerning all patients and GPs meeting the inclusion criteria, and thus eliminates selection, recall or reporting bias. All regular GPs with normal practice activity in 2009 were included, so the study was representative of general practice in Norway.

### Limitations

A major limitation is the selection of patient groups based on diagnoses set by GPs in the routine data used for remuneration purposes and not for research. However, other findings have indicated that diagnoses used in GP records are valid overall, and there are no incentives to apply incorrect diagnoses [33,34]. The diagnoses of schizophrenia found in administrative data have been shown to be valid [35]. Usually a diagnosis of schizophrenia is confirmed by specialised mental health care services after an admission, and few GPs apply the diagnosis when not justified. However, cases can have been missed for different reasons, resulting in underdiagnoses compared with the real prevalence of schizophrenia in the population. A Norwegian study estimated a point prevalence of schizophrenia in the adult population to be 0.4% [36], and internationally, a prevalence of up to 0.6% has been found [37]. Thus, the identified patients in this study probably constitute a large proportion of all patients with schizophrenia in Norway.

The prevalence of DM in the adult population based on diagnoses in GP claims data also corresponds well with earlier Norwegian prevalence estimates of 2–3% in the relevant age groups [38]. We decided to include an analysis on the role of comorbidity in GP service utilisation. However, there is limited information and a lack of precision concerning comorbidity in GP claims since most claims have only one diagnosis even if several conditions have been dealt with in the consultation.

Finally, claims may be used to maximize the income of the GPs and over-reporting of the services given cannot be ruled out. However, the claims system relies on strict control mechanisms. Fraud has serious consequences and is probably limited among GPs.

### Comparison with previous studies

The consultation rates found in this study from general practice in Norway are in line with Dutch and British studies [5,6]. In a Nordic survey from 2003, only 17% of patients with schizophrenia reported a GP contact during the last year [7]. The present register-based study indicates that this proportion has markedly increased after the establishment of a regular GP scheme. Many GPs participate in primary care teams concerning their patients, even for those who do not consult the GP in the office. This finding partly contradicts earlier findings of a low participation rate in multidisciplinary work among GPs [28].

The negative association between total FFS and the length of the patient-GP relationship supports earlier findings. Personal continuity and cumulative knowledge may lead to more efficient use of resources [39]. The study also indicated that the GPs may provide a more comprehensive service for patients with SMDs in smaller municipalities, also reported in earlier studies [7]. In addition, specialist mental health services are located mainly in larger municipalities allowing easier access for patients in these areas.

### Comorbidity

The GPs’ diagnoses indicated a 3.5 times higher prevalence of DM in the schizophrenia group compared with the total population of the same age, which corresponds to previous studies [40]. The proportion of patients with schizophrenia who also had a diagnosis of obstructive lung disease was only moderately elevated compared with the total population and may be under-recognized compared with a previous study [40]. Based on the diagnosis reported by GPs, the prevalence of hypertension and cardiovascular diseases among patients with schizophrenia is similar to population means. However, based on earlier studies, a higher prevalence of these diseases among patients with schizophrenia could be expected [15,16].

### FFS and comorbidity

Comorbidities of DM, obstructive lung diseases, or cardiovascular disease among patients with schizophrenia was associated with markedly increased utilisation of GP services than in comparator groups without schizophrenia. Also, the use of tests or procedures to monitor somatic illnesses were used equally or more often in these patients than among patients without SMD, which is in line with findings in a study of GPs in The Netherlands [23].

### Further research and implications for practice

In a recent survey, 77% of GPs in Norway stated that caring for patients with SMD was among their most meaningful tasks, and 29% wanted to spend more time on this patient group [41]. However, there might be a need for a clearer strategy and guidelines for the role of the GP in mental health care [25,35,42], and research is needed to provide a scientific basis for improvements.

GPs used the tariff code marked with the term “talking therapy” in nearly half of consultations with patients diagnosed with schizophrenia. If we accept that GPs, when using this code, take time to discuss the patient’s problems extensively, this implies that GPs in general also care about the patient’s psychosocial situation. On the other hand, most patients with schizophrenia have less than five consultations with their GPs annually. Other professions probably have the main responsibility for many of these patients, but many may have sub-optimal care from the health services in total [6,17,22]. Further studies on the GPs’ use of “talking therapy” and on their cooperation with other sections of the mental health service are needed.

In this study, the GP’s “mental health practice profile” appeared to be indicative of the amount of services given to individual patients with schizophrenia. This finding may reflect that GPs’ fields of interest result in different content of services to different patient groups [41]. This might also result in disparities in GP services for patients with SMD.

The detection rate of respiratory and cardiovascular comorbidity among patients with schizophrenia is probably too low, and indicates that Norwegian GPs should be more aware of these conditions among their SMD patients.

Finally, we cannot conclude from this study whether services provided by Norwegian GPs to patients with schizophrenia are sufficient with respect to the total burden of disease. The content and quality of services should be examined in further studies.

## Conclusion

A large proportion of patients diagnosed with schizophrenia have regular contact with their GP, providing opportunities for the GP to take care of both mental and somatic health problems among these patients. The study indicates high total GP service utilization, and the use of tests to monitor comorbid diseases is sufficient compared with similar patients without SMD.
